# Endometrioid Cancer Associated With Endometriosis: From the Seed and Soil Theory to Clinical Practice

**DOI:** 10.3389/fonc.2022.859510

**Published:** 2022-03-10

**Authors:** Alberto Farolfi, Amelia Altavilla, Luca Morandi, Laura Capelli, Elisa Chiadini, Giovanna Prisinzano, Giorgia Gurioli, Marianna Molari, Daniele Calistri, Maria Pia Foschini, Ugo De Giorgi

**Affiliations:** ^1^ Department of Medical Oncology, IRCCS Istituto Romagnolo per lo Studio dei Tumori (IRST) “Dino Amadori”, Meldola, Italy; ^2^ Department of Biomedical and Neuromotor Sciences, University of Bologna, Bologna, Italy; ^3^ Functional and Molecular Neuroimaging Unit, IRCCS Istituto delle Scienze Neurologiche di Bologna, Bologna, Italy; ^4^ Biosciences Laboratory, IRCCS Istituto Romagnolo per lo Studio dei Tumori (IRST) “Dino Amadori”, Meldola, Italy; ^5^ Unit of Anatomic Pathology, Department of Biomedical and Neuromotor Sciences, Bellaria Hospital, University of Bologna, Bologna, Italy

**Keywords:** uterine carcinoma, endometriosis, endometrioid adenocarcinoma of the endometrium, mismatch repair (MMR) deficiency, tumor dissemination

## Abstract

Endometriosis is a benign condition characterized by the presence of ectopic endometrial tissue. It is still debated whether endometriosis is a disease that can predispose to the pathogenesis of endometrial cancer outside the uterus. Deficiencies in mismatch repair (MMR) genes are a known risk factor for developing endometrioid cancer. Starting from two cases of patients with abnormal MMR endometrioid carcinoma of the uterus and synchronous endometrioid carcinoma in non-ovarian and ovarian endometriosis, we performed a somatic mutation profile and phylogenetic analysis of the lesions in order to identify if they were metastasis or primary *de novo* tumors. In the first case, we identified *de novo* activating mutations in *PIK3CA* and *KRAS* in endometrioid cancer lesions but not in endometriosis. Although the acquisition of a *de novo* mutation in *ESR1* and a decrease in mutant allele fraction (MAF) from the endometrial tumor to the localizations in the endometriosis lesions, the clonal relationship was confirmed by the limited number of heteroplasmic mutations in D-loop mitochondrial DNA region. In the other case, the clonal behavior was demonstrated by the overlap of MAF at each site. Our data support the hypothesis of a retrograde dissemination of tumor cells, moving from the primary carcinoma in the endometrium to ectopic sites of endometriosis where localizations of tumor arise.

## Introduction

Endometriosis is a relatively common disease, characterized by the presence of ectopic endometrial tissue outside the uterus (1). Endometriosis affects 10%–15% of all women of reproductive age (2) and approximately 2%–5% of postmenopausal women, representing a side effect of hormonal replacement therapy or tamoxifen treatment in this population (3). The etiology underlying endometriosis is controversial, but the processes proposed in its development closely resemble those involved in cancer metastasis (4). Ovarian endometriosis has been reported to be associated with an increased risk of epithelial ovarian cancer, representing the direct precursor of clear-cell and endometrioid ovarian carcinomas (5). However, whether extraovarian endometriosis may be an endometrioid cancer precursor remains controversial (6, 7).

Endometrial cancer is a clinically heterogeneous disease. Genomic characterization by The Cancer Genome Atlas (TCGA) classified endometrial cancers into four categories: POLE ultramutated, microsatellite instability hypermutated, copy-number low, and copy-number high (8). Another classification of endometrial cancer differentiates tumors into two subtypes: type I is characterized by a favorable prognosis and represented mostly by endometrioid adenocarcinoma, associated with an unopposed estrogen stimulation, often preceded by endometrial hyperplasia; type II has significantly poorer 5-year survival predominantly represented by non-endometrioid histology, mostly arising in an atrophic endometrium and deriving from intraepithelial carcinoma as a precancerous lesion (9). Since the evidence of activity of immune checkpoint inhibitors in patients with advanced mismatch repair (MMR)-deficient endometrial cancer (10) and considering that Lynch syndrome may account for about 3% of all endometrial cancers (11), it is recommended to screen all endometrial cancer patients with the use of immunohistochemical tests for *MLH1*, *MSH2*, *MSH6*, and *PMS2* (12).

Whether patients with endometriosis have increased risks of development of endometrioid tumors or if endometriosis is the soil where endometrioid cancer seeds grow remains unclear. In this study, we report two cases of patients with MMR-deficient endometrioid carcinoma of the uterus and synchronous endometrioid carcinoma in extraovarian and ovarian endometriosis.

## Methods

### Tissue Sample and Mismatch Repair Evaluation

Tissue samples were paraffin-embedded archival specimens. Immunohistochemistry was performed at the Pathology Unit. Antibodies against MLH1, MSH2, MSH6, and PMS2 were prediluted according to the manufacturer’s instructions. The expression of MLH1, MSH2, MSH6, and PMS2 was determined qualitatively to be retained or lost, as is standard.

Molecular analyses on genomic DNA extracted from peripheral blood were performed to search for variants in MMR (*MLH1*, *MSH2*, and *MSH6*) genes. Moreover, all coding exons and splice junctions of the *MLH1*, *MSH2*, and *MSH6* genes were analyzed. The analysis for the identification of variants and genomic rearrangements [copy number variations (CNVs)] was performed by next-generation sequencing (NGS) with Miseq-Illumina sequencer *via* commercial panel HCS_v1_1 (Sophia Genetics).

### Nucleic Acid Extraction and Quantification

Dual DNA and RNA isolation was performed from four formalin-fixed paraffin-embedded (FFPE) tissues using the Maxwell^®^ RSC DNA/RNA FFPE Kit with the Maxwell^®^ RSC Instrument. For each sample, areas were characterized by 100% tumor cells. Nucleic acid concentrations were determined by fluorometric quantitation using Qubit 4.0 Fluorimeter with Qubit dsDNA HS Assay Kit and Qubit RNA HS Assay Kit (Thermo Fisher Scientific, Inc.).

### Next-Generation Sequencing

To estimate somatic mutation profiling, NGS was performed with the “Ion Torrent Oncomine Focus Assay” for simultaneous and rapid identification of single-nucleotide variants (SNVs), short insertion and deletions (indels; 35 genes), CNVs (19 genes), and gene rearrangements (23 genes) in 52 cancer genes with therapeutic relevance:

Hotspot genes (35): AKT1, ALK, AR, BRAF, CDK4, CTNNB1, DDR2, EGFR, ERBB2, ERBB3, ERBB4, ESR1, FGFR2, FGFR3, GNA11, GNAQ, HRAS, IDH1, IDH2, JAK1, JAK2, JAK3, KIT, KRAS, MAP2K1, MAP2K2, MET, MTOR, NRAS, PDGFRA, PIK3CA, RAF1, RET, ROS1, and SMO.

CNV genes (19): ALK, AR, BRAF, CCND1, CDK4, CDK6, EGFR, ERBB2, FGFR1, FGFR2, FGFR3, FGFR4, KIT, KRAS, MET, MYC, MYCN, PDGFRA, and PIK3CA.

Fusion driver genes (23): ABL1, ALK, AKT3, AXL, BRAF, EGFR, ERBB2, ERG, ETV1, ETV4, ETV5, FGFR1, FGFR2, FGFR3, MET, NTRK1, NTRK2, NTRK3, PDGFRA, PPARG, RAF1, RET, and ROS1.

The Invitrogen SuperScript™ VILO™ cDNA Synthesis Kit (Thermo Fisher Scientific) was used for RNA reverse transcription to cDNA before library preparation.

Libraries were prepared from 10 ng DNA and 10 ng RNA (0.67 ng/µl, 15 µl) with “Oncomine Focus Assay, Chef-Ready Library” reagents on the Ion Chef™ System (Thermo Fisher Scientific).

Templating and sequencing were performed using “Ion 510™ & Ion 520™ & Ion 530™ Kit–Chef.”

For template preparation, we used Ion 520 chip (up to 5 million reads per chip, 8 samples) on the Ion Chef™ System, while sequencing was completed on the Ion Torrent S5 Plus (Thermo Fisher Scientific).

Sequencing data were analyzed using Ion Reporter™ Software that helps to identify and prioritize variants.

To define a reliable variant calling, we have considered two stringent parameters: coverage depth greater than 500× and allele frequency greater than 5%.

### Phylogenetic Trees

Phylogenetic analysis starting from different tumor populations in the Pathology Unit of Bellaria Hospital (Bologna) was performed as previously described (13, 14). In brief, mitochondrial DNA (mtDNA) D-loop region was sequenced in deep onto MiSEQ (Illumina) and processed by Geneious 9.1.8 (Biomatters Ltd., Auckland, New Zealand) to identify and annotate homoplasmic or heteroplasmic mutations. The 4 consensus sequences representative of each of the four tumor populations were joined and used to construct the phylogenetic tree using Multiple Alignment using Fast Fourier Transform (MAFFT; https://mafft.cbrc.jp/alignment/server/) with the unweighted pair group method with arithmetic mean and the Jukes-Cantor substitution model.

## Results

### Case Report 1

A 58-year-old woman was diagnosed with a grade 2 endometrioid carcinoma in August 2020. After having ruled out other neoplastic lesions with thorax and abdomen computed tomography (CT) scan and pelvic MRI, the patient underwent a total hysterectomy with bilateral annexectomy and pelvic lymphoadenectomy. During surgery, two peritoneal lesions in the rectouterine pouch were also removed. The diagnosis was of endometrioid carcinoma of the uterus grade 3, with more than 50% invasion of the myometrial wall thickness, 5.2 cm in greatest dimension, with infiltration of the cervix stroma, without lymphovascular invasion, and no pelvic lymph node metastases. The two peritoneal nodules were diagnosed as duplex localization of endometrioid carcinoma grade 3 in endometriosis lesions. In the left ovary, a localization of endometriosis with another endometrioid carcinoma grade 3 lesion was diagnosed [stage pT3aN0M1, Fédération Internationale de Gynécologie et d’Obstétrique (FIGO IVB)]. Using immunohistochemistry, tumor cells were shown to have a normal pattern of expression of p53, MSH2, and MSH6, whereas MLH1 and PMS2 were lost. For this reason, molecular analysis was performed to search for variants in MMR genes on genomic DNA extracted from peripheral blood. The regions of *MLH1*, *MSH2*, and *MSH6* genes analyzed did not show specific alterations nor partial or complete genomic rearrangements (CNVs).

Five cycles of chemotherapy with carboplatin and paclitaxel were administered. The sixth cycle was omitted for hematologic toxicity (grade 3 anemia and grade 3 thrombocytopenia). Patient then received adjuvant external pelvic radiotherapy at a dose of 45 Gray. Until now, patient is disease-free.

#### Molecular Profiling

**Figure 1:**
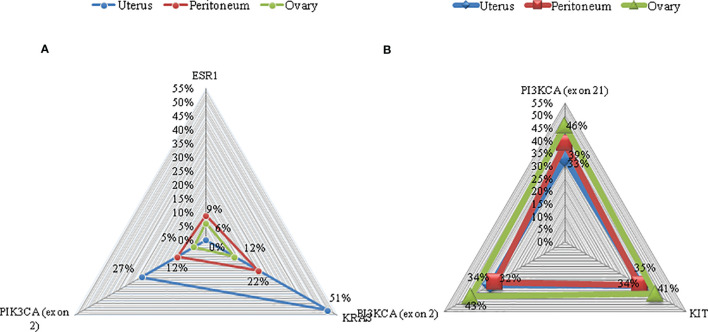
Mutations on representative cancer-associated genes of case 1 **(A)** and case 2 **(B)**. Each color represents a different sample. Triangles represent the mutant allele frequency (MAF), corresponding to those of the vertical axis.

In order to identify if the endometrioid carcinoma lesions in the peritoneum and in the ovary were synchronous tumors that arose in extraovarian and ovarian endometriosis tissue or metastasis, a molecular profiling was performed. Targeted sequencing in primary uterine tumor revealed an activating *PIK3CA* hotspot exon 2 mutation (c.263G>A) with an allelic ratio of 27% and a *KRAS* exon 2 hotspot mutation (c.34G>A) with an allelic ratio of 51%. Comparable mutations were found in the tumor lesion of the left paracolpium: *PIK3CA* exon 2 mutation (c.263G>A) and a *KRAS* exon 2 mutation (c.34G>A) with an allelic ratio of 12% and 22%, respectively. An activating *ESR1* hotspot exon 8 mutation (c.1607T>G) with an allelic ratio of 9% was also identified. The same mutations were detected in the tumor lesion of the left ovary with less allelic ratio: *PIK3CA* exon 2 mutation (c.263G>A), *KRAS* exon 2 mutation (c.34G>A), and *ESR1* hotspot exon 8 mutation (c.1607T>G) with an allelic ratio of 5%, 12%, and 6%, respectively (**Figure 1**). No hotspot mutations were identified (with a sensitivity of detection of 1%) in the endometriosis nodules without tumor lesions.

**Figure 2:**
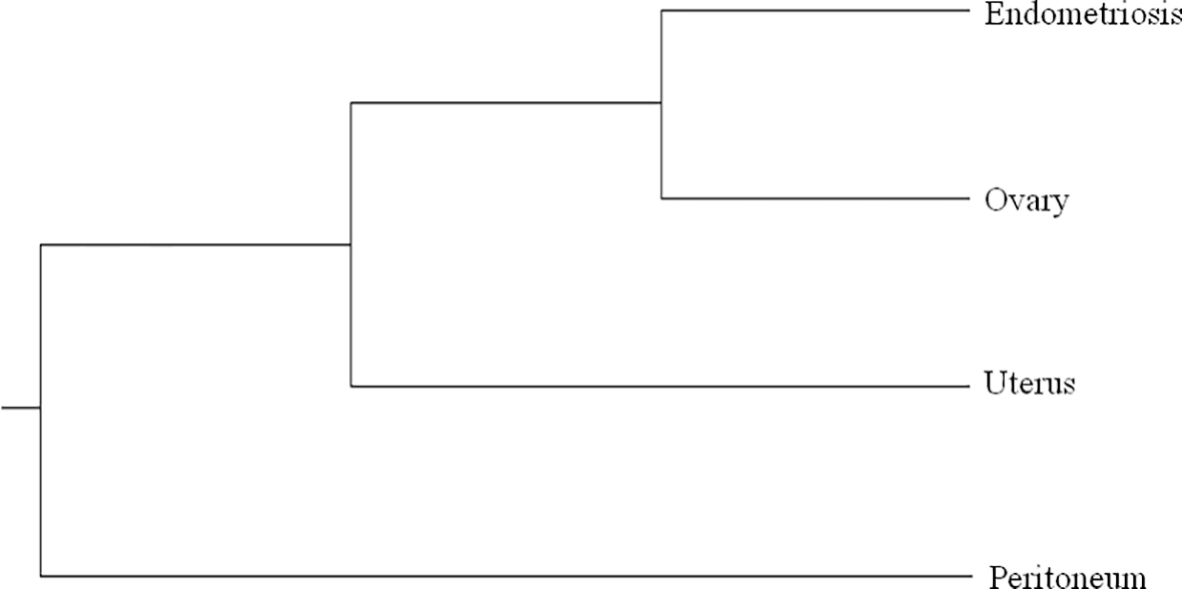
Phylogenetic tree based on Multiple Alignment using Fast Fourier Transform (MAFFT) with the unweighted pair group method with arithmetic mean and the Jukes–Cantor substitution model. All the samples of case 1 are very close to each other, as we found a limited number of mutations in heteroplasmy, 4 of which in the sample of the peritoneum and 2 in endometriosis sample.

The phylogenetic relationship of multiple samples from different tumor regions was also evaluated by sequencing D-loop mtDNA region. We found 6 heteroplasmic mutations, 4 of which were present only in specimen A1, while 2 were present only in specimen A12. The phylogenetic tree is shown in **Figure 2**. All samples were close to each other, indicating a possible clonal relationship among them.

### Case Report 2

In April 2021, a 60-year-old woman was diagnosed with a grade 2 endometrioid carcinoma. Chest and abdominal contrast-enhanced CT scan revealed no metastatic lesions. Patient underwent a total hysterectomy with bilateral annexectomy and low anterior resection of rectum with colostomy. During surgery, a single peritoneal lesion in the rectouterine pouch was biopsied. The surgical specimen of the uterus showed a 50 × 35 mm polypoid tumor. Histopathological diagnosis was grade 2 endometrial carcinoma with more than 50% invasion of the myometrial wall thickness, infiltration of perirectal fat, and lymphovascular invasion. In the left ovary, a localization of endometriosis with another endometrioid carcinoma grade 2 lesion was diagnosed. The peritoneal lesion was positive for endometrioid carcinoma grade 2 (stage pT3bNXM1, FIGO IVB). Tumor cells were shown to have a normal pattern of expression of p53 and MLH1 and PMS2 positives at immunohistochemistry. For this reason, molecular analysis was performed to search for variants in MMR genes on genomic DNA extracted from peripheral blood. The regions of *MLH1* and *MSH6* genes analyzed did not show any specific alteration nor partial or complete genomic rearrangements (CNVs), while *MLH2* gene showed a non-classified variant (c.728G>A;p).

Six cycles of chemotherapy with carboplatin and paclitaxel were administered, and adjuvant external pelvic radiotherapy is ongoing. Until now, patient is disease-free.

#### Molecular Profiling

Also in this patient, a molecular profiling was performed to identify if the endometrioid carcinoma lesions in the left ovary were synchronous tumors that arose in extraovarian and ovarian endometriosis tissue or metastasis.

Targeted sequencing in primary uterine tumor revealed an activating *PIK3CA* hotspot exon 2 mutation (c.263G>A) with an allelic ratio of 34% and hotspot exon 21 mutation (c c.3129G>T) with an allelic ratio of 33%. Furthermore, a variant of uncertain significance of *cKIT* exon 8 mutation (c.1264G>A) with an allelic ratio of 35% was found. Comparable mutations were found in the peritoneum tumor localization: *PIK3CA* exon 2 mutation (c.263G>A) and exon 21 mutation (c c.3129G>T) with an allelic ratio of 32% and 39%, respectively; *cKIT* exon 8 mutation (c.1264G>A) with an allelic ratio of 34%. Again, tumor lesion of the left ovary presented *PIK3CA* exon 2 mutation (c.263G>A) and exon 21 mutation (c c.3129G>T) with an allelic ratio of 43% and 46%, respectively; *cKIT* exon 8 mutation (c.1264G>A) with an allelic ratio of 41%.

## Discussion

Although endometriosis is considered to be a benign lesion, malignant transformation in endometriosis-related ovarian neoplasms is possible (15). In recent years, different studies demonstrated that endometriosis (ovarian and extraovarian endometriotic lesions) harbors somatic mutations in cancer driver genes (6, 16). Since somatic mutations are a widespread event in “normal” tissue (endometrial samples included) and most endometriotic lesions harboring cancer-associated genes do not necessarily lead to malignant transformation (17), environmental features protective against malignant transformation or that buffer the effects of such mutations may prevent the progression from endometriosis to gynecologic cancers (16). Moreover, it was hypothesized that alterations in MMR genes might be involved in the malignant transformation of endometriotic lesions (7).

**Figure 3:**
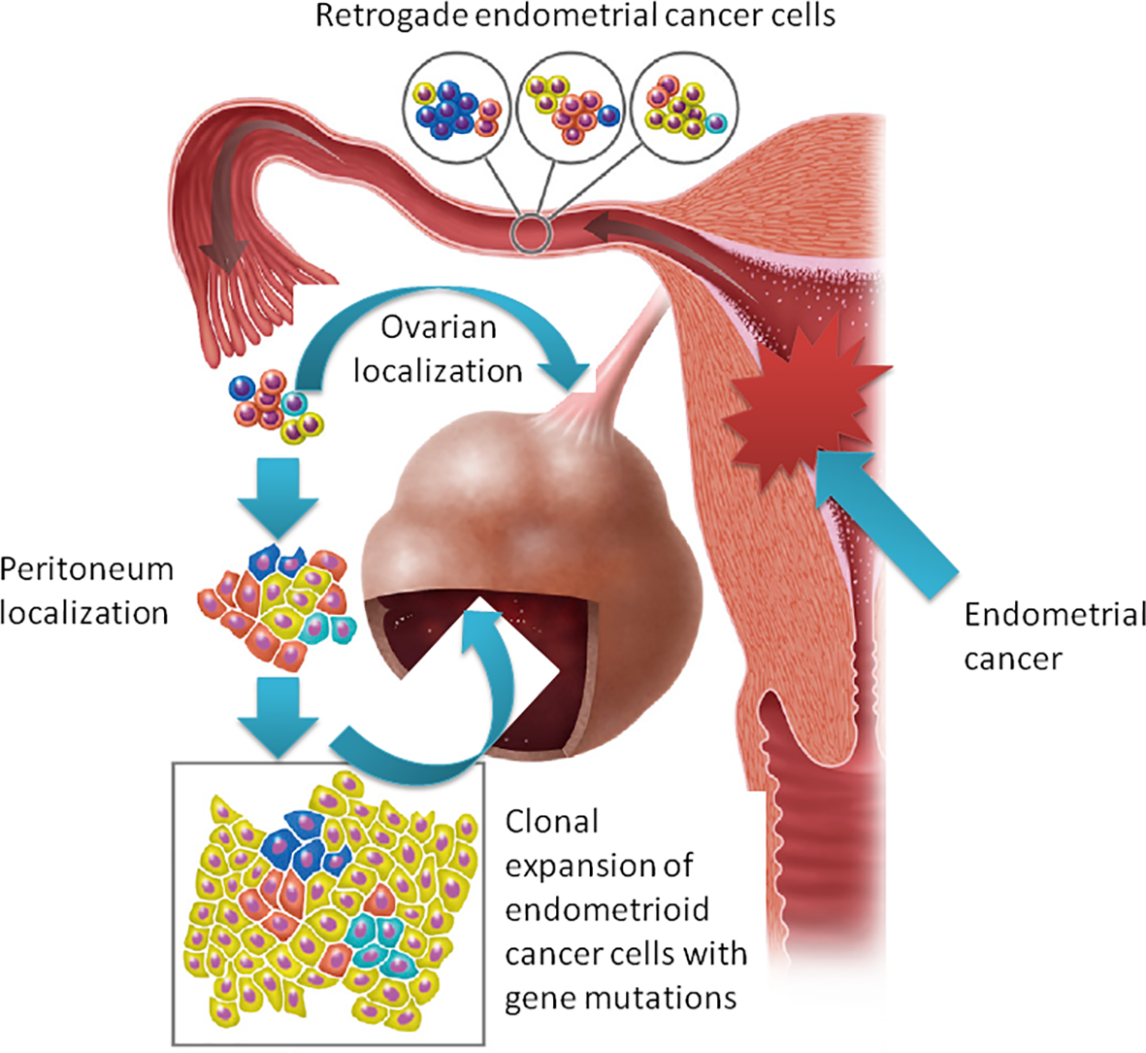
The “seed and soil” hypothesis: retrograde flow of endometrial cells already harboring cancer-associated mutation moving from the primary carcinoma in the endometrium to ectopic sites of endometriosis where tumor localizations arise.

With these premises, when we faced two similar cases of patients with endometrioid carcinoma of the uterus with MMR deficiency and synchronous endometrioid carcinoma in extraovarian and ovarian endometriosis, the question was whether the latter was subsequent to malignant transformation of endometriotic lesions or to secondary localization. To solve this dilemma, we performed a molecular profile of the cancer and endometriotic lesions of the two cases. In the first case, we identified *de novo* activating *PIK3CA* and *KRAS* mutations in endometrioid cancer lesions but not in endometriosis. This was an unexpected result, since *KRAS* mutations were associated with endometriosis sustainability (6, 18). Moreover, case 1 showed a decrease in mutant allele fraction (MAF) and the acquisition of a *de novo* mutation (e.g., *ESR1*) from primary tumor to distant lesions. For this reason, we performed a phylogenetic relationship of multiple samples from different tumor regions sequencing D-loop mtDNA region. According to this analysis, a limited number of heteroplasmic mutations were found showing a close phylogenetic distance among different tumor populations, demonstrating that peritoneal and ovarian carcinomas are derived from the same endometrial ancestor clone that migrated and settled in endometriotic lesions where the endometrioid tumor arose (**Figure 3**). In case 2., the MAF was almost the same in all the different lesions, supporting the idea of the clonal relationship between the different tumor localizations.

Both patients had MMR-deficient tumors. MMR alterations may result in genetic tumor heterogeneity (as seen in case 1) but could also be a risk factor for cancer development in endometriotic heterotopic tissue (7, 19). However, our data support the hypothesis of a retrograde dissemination of tumor cells, moving from the primary carcinoma in the endometrium to ectopic sites of endometriosis where localizations of tumor may arise, reinforcing literature data on the origin of endometriosis possibly following retrograde menstruations (20).

A brief comment on *ESR1* found in one of our cases is needed. Our results suggest that the *ESR1* mutation was a *de novo* mutation that arose without the selective pressure of a hormonal treatment. This observation suggests that *ESR1* may be a biomarker of progression rather than a predictor of endocrine therapy resistance (21) in endometrial cancer. Therefore, characterization only at the time of progression to hormone therapy may be inadequate (22) because *ESR1*-mutated tumor cells may be the result of a clonal expansion of endometrioid cancer cells primarily refractory to hormonal treatment.

In conclusion, endometrioid tumors, especially if an alteration in the MMR genes occurs, represent a heterogeneous disease, and the acquisition of new mutations is an event that needs to be considered. Although cancer-associated mutations are frequently observed in endometriosis, our study demonstrated that endometriosis-associated carcinoma may arise from cancer seeds that implant in this permissive soil moving in a retrograde way rather than a direct malignant transformation. How this disease has to be treated and considered (e.g., is really a stage IV endometrial cancer?) is still a matter of debate.

## Data Availability Statement

The datasets presented in this study can be found in online repositories. The names of the repository/repositories and accession number(s) can be found in the article/supplementary material.

## Ethics Statement

Ethical review and approval was not required for the study on human participants in accordance with the local legislation and institutional requirements. The patients/participants provided their written informed consent to participate in this study.

## Author Contributions

AF, LM, MPF, and UDG: conception and planning of the work. AF, AA, LC, EC, GP, GG, MM, and DC: acquisition and analysis of the data. AF, AA, LM, and UDG: interpretation of the data. AF, AA, LM, and GG: drafting and/or critical revision of the article for important intellectual content. All the authors read and approved the final submitted version of the article.

## Conflict of Interest

The authors declare that the research was conducted in the absence of any commercial or financial relationships that could be construed as a potential conflict of interest.

## Publisher’s Note

All claims expressed in this article are solely those of the authors and do not necessarily represent those of their affiliated organizations, or those of the publisher, the editors and the reviewers. Any product that may be evaluated in this article, or claim that may be made by its manufacturer, is not guaranteed or endorsed by the publisher.

## References

[1] Giudice LC. Clinical Practice. Endometriosis N Engl J Med (2010) 362:2389–98. doi: 10.1056/NEJMcp1000274 PMC3108065 20573927

[2] Giudice LC Kao LC. Endometriosis. Lancet (2004) 364:1789–99. doi: 10.1016/S0140-6736(04)17403-5. 15541453

[3] Parasar P Ozcan P Terry KL. Endometriosis: Epidemiology, Diagnosis and Clinical Management. Curr Obstet Gynecol Rep (2017) 6:34–41. doi: 10.3390/diagnostics10030134 29276652 PMC5737931

[4] Bulun SE. Endometriosis. N Engl J Med (2009) 360:268–79. doi: 10.1056/NEJMra0804690 19144942

[5] Ogawa S Kaku T Amada S Kobayashi H Hirakawa T Ariyoshi K. Ovarian Endometriosis Associated With Ovarian Carcinoma: A Clinicopathological and Immunohistochemical Study. Gynecol Oncol (2000) 77:298–304. doi: 10.1006/gyno.2000.5765 10785482

[6] Anglesio MS Papadopoulos N Ayhan A Nazeran TM Noë M Horlings HM. Cancer-Associated Mutations in Endometriosis Without Cancer. N Engl J Med (2017) 376:1835–48. doi: 10.1056/NEJMoa1614814 PMC5555376 28489996

[7] Fuseya C Horiuchi A Hayashi A Suzuki A Miyamoto T Hayashi T. Involvement of Pelvic Inflammation-Related Mismatch Repair Abnormalities and Microsatellite Instability in the Malignant Transformation of Ovarian Endometriosis. Hum Pathol (2012) 43:1964–72. doi: 10.1016/j.humpath.2012.02.005 22626277

[8] Cancer Genome Atlas Research Network Kandoth C Schultz N Cherniack AD Akbani R Liu Y. Integrated Genomic Characterization of Endometrial Carcinoma. Nature (2013) 497:67–73. doi: 10.1038/nature12113 23636398 PMC3704730

[9] Malik TY Chishti U Aziz AB Sheikh I. Comparison of Risk Factors and Survival of Type 1 and Type II Endometrial Cancers. Pak J Med Sci (2016) 32:886–90. doi: 10.12669/pjms.324.9265 PMC5017096 27648033

[10] Oaknin A Tinker AV Gilbert L Samouëlian V Mathews C Brown J. Clinical Activity and Safety of the Anti-Programmed Death 1 Monoclonal Antibody Dostarlimab for Patients With Recurrent or Advanced Mismatch Repair-Deficient Endometrial Cancer: A Nonrandomized Phase 1 Clinical Trial. JAMA Oncol (2020) 6:1766–72. doi: 10.1001/jamaoncol.2020.4515 PMC7530821 33001143

[11] Ryan N Morris J Green K Lalloo F Woodward ER Hill J. Association of Mismatch Repair Mutation With Age at Cancer Onset in Lynch Syndrome: Implications for Stratified Surveillance Strategies. JAMA Oncol (2017) 3:1702–6. doi: 10.1001/jamaoncol.2017.0619 PMC5824283 28772289

[12] Murali R Delair DF Bean SM Abu-Rustum NR Soslow RA. Evolving Roles of Histologic Evaluation and Molecular/Genomic Profiling in the Management of Endometrial Cancer. J Natl Compr Canc Netw (2018) 16:201–9. doi: 10.6004/jnccn.2017.7066 PMC6639790 29439179

[13] Gabusi A Gissi DB Tarsitano A Asioli S Marchetti C Montebugnoli L. Intratumoral Heterogeneity in Recurrent Metastatic Squamous Cell Carcinoma of the Oral Cavity: New Perspectives Afforded by Multiregion DNA Sequencing and Mtdna Analysis. J Oral Maxillofac Surg (2019) 77:440–55. doi: 10.1016/j.joms.2018.09.014 30321517

[14] Gissi DB Tarsitano A Leonardi E Gabusi A Neri F Marchetti C. Clonal Analysis as a Prognostic Factor in Multiple Oral Squamous Cell Carcinoma. Oral Oncol (2017) 67:131–7. doi: 10.1016/j.oraloncology.2017.02.017 28351567

[15] Wei JJ William J Bulun S. Endometriosis and Ovarian Cancer: A Review of Clinical, Pathologic, and Molecular Aspects. Int J Gynecol Pathol (2011) 30:553–68. doi: 10.1097/PGP.0b013e31821f4b85 PMC4130217 21979592

[16] Suda K Nakaoka H Yoshihara K Ishiguro T Tamura R Mori Y. Clonal Expansion and Diversification of Cancer-Associated Mutations in Endometriosis and Normal Endometrium. Cell Rep (2018) 24:1777–89. doi: 10.1016/j.celrep.2018.07.037 30110635

[17] Guo SW. Cancer-Associated Mutations in Endometriosis: Shedding Light on the Pathogenesis and Pathophysiology. Hum Reprod Update (2020) 26:423–49. doi: 10.1093/humupd/dmz047 32154564

[18] Cheng CW Licence D Cook E Luo F Arends MJ Smith SK. Activation of Mutated K-Ras in Donor Endometrial Epithelium and Stroma Promotes Lesion Growth in an Intact Immunocompetent Murine Model of Endometriosis. J Pathol (2011) 224:261–9. doi: 10.1002/path.2852 21480232

[19] Bennett JA Pesci A Morales-Oyarvide V Da Silva A Nardi V Oliva E. Incidence of Mismatch Repair Protein Deficiency and Associated Clinicopathologic Features in a Cohort of 104 Ovarian Endometrioid Carcinomas. Am J Surg Pathol (2019) 43(2):235–43. doi: 10.1097/PAS.0000000000001165 30256257

[20] Sampson JA. Metastatic or Embolic Endometriosis, Due to the Menstrual Dissemination of Endometrial Tissue Into the Venous Circulation. Am J Pathol (1927) 3:93–110.43. 19969738 PMC1931779

[21] Robinson DR Wu YM Vats P Su F Lonigro RJ Cao X. Activating ESR1 Mutations in Hormone-Resistant Metastatic Breast Cancer. Nat Genet (2013) 45(12):1446–51. doi: 10.1038/ng.2823 PMC4009946 24185510

[22] Morel A Masliah-Planchon J Bataillon G Becette V Morel C Antonio S. *De Novo* ESR1 Hotspot Mutation in a Patient With Endometrial Cancer Treated With an Aromatase Inhibitor. JCO Precis Oncol (2019) 3:PO.18.00398. doi: 10.1200/PO.18.00398 32914015 PMC7446375

