# Correction: Endometrioid cancer associated with endometriosis: from the seed and soil theory to clinical practice

**DOI:** 10.3389/fonc.2026.1867620

**Published:** 2026-06-25

**Authors:** Alberto Farolfi, Amelia Altavilla, Luca Morandi, Laura Capelli, Elisa Chiadini, Giovanna Prisinzano, Giorgia Gurioli, Marianna Molari, Daniele Calistri, Maria Pia Foschini, Ugo De Giorgi

**Affiliations:** 1 Department of Medical Oncology, IRCCS Istituto Romagnolo per lo Studio dei Tumori (IRST) “Dino Amadori”, Meldola, Italy; 2 Department of Biomedical and Neuromotor Sciences, University of Bologna, Bologna, Italy; 3 Functional and Molecular Neuroimaging Unit, IRCCS Istituto delle Scienze Neurologiche di Bologna, Bologna, Italy; 4 Biosciences Laboratory, IRCCS Istituto Romagnolo per lo Studio dei Tumori (IRST) “Dino Amadori”, Meldola, Italy; 5 Unit of Anatomic Pathology, Department of Biomedical and Neuromotor Sciences, Bellaria Hospital, University of Bologna, Bologna, Italy

**Keywords:** uterine carcinoma, endometriosis, endometrioid adenocarcinoma of the endometrium, mismatch repair (MMR) deficiency, tumor dissemination

There was a mistake in the caption of **Figure 3** as published, acknowledging that it was modified from the previously cited 2018 Cell Reports paper. The corrected caption of **Figure 3** appears below.

**Figure 3:**
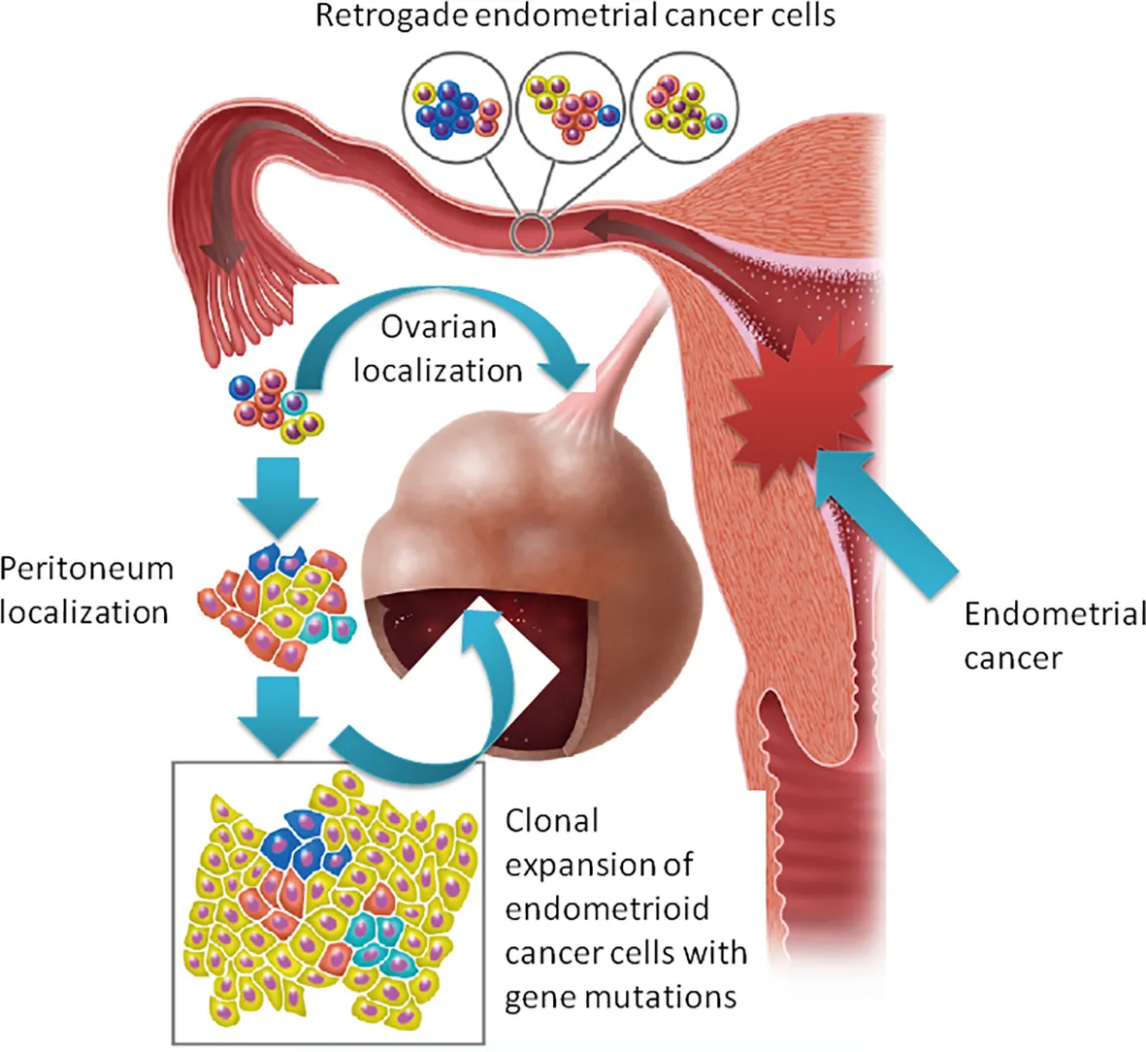
The “seed and soil” hypothesis: retrograde flow of endometrial cells already harboring cancer-associated mutation moving from the primary carcinoma in the endometrium to ectopic sites of endometriosis where tumor localizations arise. Modified from Suda K et al. (16).

The “seed and soil” hypothesis: retrograde flow of endometrial cells already harboring cancer-associated mutation moving from the primary carcinoma in the endometrium to ectopic sites of endometriosis where tumor localizations arise. Modified from Suda K et al. (16).

The original version of this article has been updated.

